# Recent Insights into Mucinous Ovarian Carcinoma

**DOI:** 10.3390/ijms19061569

**Published:** 2018-05-24

**Authors:** Francesca Ricci, Roberta Affatato, Laura Carrassa, Giovanna Damia

**Affiliations:** IRCCS-Istituto di Ricerche Farmacologiche Mario Negri, Laboratory of Molecular Pharmacology, Via Giuseppe La Masa 19, 20156 Milan, Italy; roberta.affatato@marionegri.it

**Keywords:** mucinous epithelial ovarian cancer (mEOC), platinum-based therapy, KRAS

## Abstract

Ovarian mucinous tumors represent a group of rare neoplasms with a still undefined cell of origin but with an apparent progression from benign to borderline to carcinoma. Even though these tumors are different from the other histological subtypes of epithelial ovarian neoplasms, they are still treated with a similar chemotherapeutic approach. Here, we review its pathogenesis, molecular alterations, (differential) diagnosis, clinical presentation and current treatment, and how recent molecular and biological information on this tumor might lead to better and more specific clinical management of patients with mucinous ovarian carcinoma.

## 1. Epithelial Ovarian Cancer: A Complex Disease Comprising Different Entities

Epithelial ovarian cancer (EOC) is a major cause of morbidity and mortality among gynecological malignancies [1]. The current standard treatment includes primary optimal debulking surgery followed by adjuvant platinum-based chemotherapy. Despite significant advances in surgery and chemotherapy over the past decades (about 70% of total patients are responsive to the treatment), the five-year survival rate for advanced stages is still low at approximately 25% [2]. 

EOC has been classified into five histological subtypes: high-grade serous carcinoma (HGSC), clear-cell carcinoma, endometrioid carcinoma, mucinous carcinoma, and low-grade serous carcinoma [3]. Although recent data strongly indicate that these subtypes differ in the site of origin, molecular signatures, response to therapy, and prognosis, all of them are still receiving front-line platinum-based therapy. 

More than a decade ago, Shih and Kurman proposed a dualistic model for EOC, based on clinico-pathological and molecular studies classifying EOC as type I and type II [4]. Type I carcinomas account for 10% of all the EOCs, are generally low-grade and include different histotypes: low-grade endometrioid, clear-cell, mucinous, and low-grade serous ovarian carcinoma. When they are confined to the ovary, they have a good prognosis, but at advanced stages, the outcome is poor because they are generally resistant to chemotherapy. Type II tumors account for the majority of all EOCs (about 90%), are generally high-grade serous, develop rapidly, are highly aggressive, and in most cases, present in an advanced stage [5,6]. These two types of EOC also present different molecular features. Generally, type I tumors are genetically stable, display a wild type (wt) *TP53*, and have alterations in the *PIK3CA*, *PTEN*, *KRAS*, *ERK,* and *ARID1A* genes. In contrast, type II tumors are genetically unstable and express a mutated *TP53,* and more than 50% of the cases harbor defects in the homologous recombination pathway, the so-called *BRCAness* phenotype [5,7,8]. 

The present review focuses on the rare mucinous epithelial ovarian carcinoma (mEOC) subtype, describing its clinical and molecular characteristics and the available treatments. 

### 1.1. Mucinous Ovarian Tumors: Clinical Considerations

Mucinous ovarian tumors can be classified as benign, borderline or malignant and can be further classified into invasive and noninvasive. Mucinous cystadenomas are considered benign, non-invasive intraepithelial carcinomas that display cellular atypia but no stroma invasion [9], while invasive mucinous ovarian carcinomas (mEOC) display stromal invasion of 5 mm [10,11]. Mucinous ovarian carcinomas present multicystic tumors with a huge amount of intracellular mucin (present in ≥50% of the cytoplasm) in more than 90% of tumor cells and contain little extracellular mucin [11]. These tumors generally grow as cystic gland-forming neoplasms, different from the ones originating from the gastrointestinal tract that are characterized by huge amounts of extracellular mucin [11]. The intestinal type is the most common subtype of mEOC and often exhibits a continuum of differentiation from benign through borderline to malignant, requiring great histological expertise for a correct diagnosis [12]. 

In addition, in contrast to other ovarian carcinoma histotypes, invasive mEOC harbors foci of benign or atypical epithelium with the same tumor *KRAS* mutations [13]. These data suggest a multistep progression model, with a *KRAS* mutation/alteration being an early somatic event. 

As nicely detailed by Kelemen et al. [11], still to be defined is the cell of origin undergoing transformation as no mucin-secreting cells have been described in a normal ovary. It has been suggested that mEOC could arise from the endocervical subtype foci of Müllerian metaplasia on the surface epithelium or from cortical inclusion cysts [11]. The expression profiles of a small number of mEOC are more closely related to those of colonic epithelium or colorectal carcinomas, suggesting a similar pathogenesis [14]. At a genetic level, mEOCs are not associated with *BRCA1* or *BRCA2* mutations. Recently, a genome-wide association study described three new genetic loci specifically associated with the risk of developing mEOC by comparing genotypes from 1644 women with mEOC and 21,693 unaffected controls [15]. One of the variants discovered is in a region that also predisposes to high-grade serous ovarian carcinoma (HGSOC) (near the *HOXD9* gene), indicating a possible shared role for this gene in the oncogenesis of both types of EOC; the other two loci (near the *INLF3* and *PAX8* genes) have been implicated in colorectal cancer. In addition, in a study involving 25,509 cases of ovarian carcinoma and 40,941 controls aimed at identifying new susceptibility loci in different histotypes of ovarian carcinoma, another two loci were found associated with mEOC (one at 3q22.3 and the other at 9q31.1) [16]. Recently, in a large pooled analysis of more than 1.3 million women from 21 studies that included 5584 EOCs (and specifically 331 women with mEOC), 14 putative risk factors were studied in relation to the different ovarian cancer subtypes [17]. Most of the factors studied were heterogeneous across the different tumor subtypes, and each subtype displayed a unique pattern of risk factor association. Smoking was associated with an increased risk of mEOC (RR, 0.72; 95% CI, 0.55 to 0.94), while oral contraceptive therapy was not. These data corroborated published data from smaller studies [18]. 

The diagnostic developments of the last decade, such as WT1 as a specific biomarker for serous differentiation [19] and the recognition of type I (low-grade carcinomas) and type II (high-grade carcinomas) as distinct pathological entities, have led to a shift in the histological diagnosis of ovarian carcinoma [20]. However, in contrast to the high-grade endometrial and serous carcinomas [21], mEOCs still lack immunohistochemical algorithms with “positive markers”. In addition, primary mEOCs have to be distinguished from metastatic tumors to the ovary, as originally suggested by Hart [22]. Metastasis from the gastrointestinal tract (colon, appendix, pancreas) and of endometrial and endocervical origin display considerable morphological overlap that makes differential diagnosis difficult, especially in advanced disease [11]. An accurate diagnosis of primary mEOC or metastatic disease is mandatory for different therapeutic approaches. Recently, an immunohistochemical profile of 36 mEOCs with many different markers has been carried out, and the typical mEOC was found to be CDK7+, with diffuse positive CK20 and CDX2 coexpression, while for SABT2, WT1, estrogen, and progesterone receptors, which are generally positive in HGSO, expressions were found to be negative [23] (Table 1). The differential diagnosis between primary mEOC and mucinous metastasis from other organs also relies on other clinical characteristics including bilaterality, surface involvement, signet ring cell presence, and lymph vascular invasion, which are more common in metastases and quite rare in primary mEOC [11,24] while tumor size 10 cm with a coexistent borderline and Brenner or dermoid tumors are clinical characteristics suggestive of primary mEOC [25], as recently reviewed [26]. It has also been reported that mucinous ovarian neoplasms associated with pseudomyxoma peritonei are now considered derived from appendiceal primary tumors [27]. 

Historically, the incidence of mEOC was reported to be around 7–14% [32,33], but more recent studies applying improved histopathology techniques and with a greater understanding of the biology and clinical history of ovarian carcinoma strongly suggest that its incidence is less common than previously thought, and that it is much closer to 3% [7,12,34].

Mucinous neoplasms generally occur in young women and are diagnosed at an early stage, with 83% being diagnosed at stage I and only 17% at stage II or higher [7]. In a retrospective study [35], patients diagnosed with mucinous adenocarcinoma between 1998 and 2003 were re-evaluated through a central pathological review. Based on a well-defined histological criteria, of the 189 patients considered, 151 were reclassified as mucinous tumors of the ovary, 64 as mucinous invasive adenocarcinomas, 45 as mucinous intraepithelial carcinomas, 42 as mucinous tumor borderline malignancies, 13 as metastatic mucinous carcinomas, and 25 as other histological subtypes. Again, these data illustrate how the diagnosis of mucinous invasive adenocarcinoma is indeed challenging. 

The availability of better diagnostic tools has led to improved histological diagnosis of EOC and to increasing evidence that the different histotypes have different biological behaviors and different patterns of sensitivity to chemotherapy (platinum-based). This is particularly true for mEOC. In 2004, Hess et al. noted that women with advanced mucinous tumors had a worse prognosis than other histological subtypes [36]; in fact, the median overall survival (OS) for women with mucinous tumors was only 12 months compared to nearly 37 months for those with nonmucinous malignancies. Stage III/IV mEOC were reported to have a much worse prognosis than HGSOC patients, with a median overall survival (OS) of 14.6 months vs. 40.6 months [37]. Bamias et al., in a retrospective study involving 367 serous, 24 mucinous, and 29 clear-cell tumors found that mEOC was the histological subtype with the worst median OS [30]. The median OS for each histological subtype was 47.7 months for HGSOC, 15.4 months for mucinous, and 36.6 months for clear-cell carcinomas. A retrospective study also showed that patients with mucinous ovarian tumors had a shorter overall survival compared to serous carcinomas, with a median survival of 14.8 and 45.1 months for mEOC and HGSOC, respectively [38]. Schiavone et al. [33] reported that women with mucinous tumors were somewhat younger, with a median age of 57 years compared to 63 years for those with serous epithelial malignancies. Among women at stage I, survival was similar for those with mucinous and serous neoplasms. However, for stage III patients, tumor histology had a strong impact on survival, and women with mucinous tumors were 55% more likely to die from their disease than those with the serous histotype (5 year survival 10.2% vs. 20.3%) [33]. 

As a whole, these data support the fact that stage III/IV mEOCs have a poor prognosis and worse than Stage III/IV HGSOCs. There is also evidence that the response rate of mEOC to standard platinum-based therapy is much lower than for HGSOC. In a retrospective analysis of 27 patients with stage III/IV mEOC, the response rate to a platinum-based therapy was 26% compared to 63% in two matched controls with non-mEOC identified for each patient [36]. Similarly, Pectasides et al. showed that in a retrospective analysis with a total of 141 patients, the overall response rates for mEOC were 38.5% and 70% for HGSOC (47 mEOC and 94 HGSOC) [28]. 

### 1.2. mEOC: Molecular Features

While HGSOCs have a 99% frequency of *TP53* mutations, only 16–52% of mEOC were reported to harbor these mutations [39,40]. Moreover, while approximately 25% of HGSOCs are associated with either germline or somatic *BRCA1/2* mutations, mucinous ovarian carcinomas are not [41,42,43]. 

The predominant mutation in mEOC is *KRAS*, which has been reported in 40–50% of the patients [11,44,45]. In an analysis of normal ovary, benign, and malignant mucinous ovarian tumors, the frequencies of *KRAS* mutations were 0%, 57%, and 76% respectively, suggesting that it might play a role in the progression from benign to malignant phenotypes [46]. *ERBB2* (human epidermal growth factor receptor 2, HER2) gene amplification has been reported in approximately 20–30% of invasive mucinous ovarian cancers and 6% of mucinous borderline ovarian carcinomas [47,48,49]. Concurrent aberrant ERBB2 and KRAS signaling has been observed in approximately 11%, suggesting that acquired *ERBB2* amplification is subsequent to the *KRAS* activating mutation [49,50]. Conversely, *KRAS* mutations and *c-MYC* amplifications, which are also common in mEOC, are mutually exclusive [39,50,51,52].

mEOC has been associated with a homozygous loss of the cyclin-dependent kinase inhibitor 2A (*CDKN2A*) locus [39,52]. Recently, IMP3 (U3 small nucleolar ribonucleoprotein) upregulation has been associated with mucinous ovarian tumor progression (from borderline tumors to invasive mEOC) [53]. Mucinous ovarian cancers were sporadically shown to harbor mutations in ring finger protein 43 (RNF43), phosphatidylinositol-4,5-bisphosphate 3-kinase catalytic subunit alpha, phosphatase and tensin homolog, cadherin 1, E74-like ETS (E-twenty-six) transcription factor 3 (ELF3), AT-rich interaction domain 1A, GNAS (Guanine Nucleotide Binding Protein G Protein, Alpha Stimulating Activity) complex locus, G protein subunit alpha 11 (GNA11), forkhead box L2, FGFR2, serine/threonine kinase 11 (*STK11*), *β-catenin* (also known as *catenin β-1*, *CTNNB1*), and SMAD family member 4 (*SMAD4*) [39,50,54,55,56]. 

Recently, a combination of tumor genome sequencing, protein expression, gene amplification, and RNA fragment analysis was performed on 304 cases of mEOC submitted to Carls Life Sciences between 2009 and 2014 [56]. The following mutational profile was found: frequent alterations in the MAP kinase pathway (49% mutations in *KRAS* and 3.5% in *BRAF*); less frequent alterations in *PIK3CA* in 12%, and *PTEN* in 6%; *cMET* overexpression (in 33% of cases) but no *cMET* gene amplification; *TP53* mutation (37%); *EGFR* gene amplification by FISH (in 50% of which 57% had EGFR overexpression by immunohistochemistry); and *HER2* gene amplification (in 11% by FISH). Lastly, PD-1 positivity was observed in 43% of tumor infiltrating lymphocytes, and PD-L1 was positive in 14% of the cases [56,57]. Src kinase has been recently reported to be overexpressed in mEOCs [58,59].

## 2. Therapeutic Approaches in mEOC

### 2.1. Chemotherapy

mEOC differs from other types of epithelial ovarian cancer in its pathogenesis, pathologic characteristics, molecular signature, and clinical behavior, and this might explain the different response to current standard-of-care therapeutic regimens compared to HGSOC. Indeed, if we exclude stage I mEOC, with its excellent prognosis of a 90% five-year survival with surgery alone [29,60], many studies have demonstrated markedly inferior outcomes for mEOC compared with HGSOC for advanced diseases, with platinum-based chemotherapy chemoresistance being the main reason for the inferior outcomes in advanced mEOC. 

mEOC cell lines had intrinsic platinum resistance [61], as well as resistance to carboplatin and to taxanes as single agents. As reviewed in detail in [26], many retrospective studies have reported the much lower response rates to platinum-based chemotherapy for mEOC compared with HGSOC [26,29,61]. What remain unclear are the reasons for the lower chemosensitivity of mEOC. It has been recently reviewed that altered mucin expression has been involved in chemoresistance in colon and breast cancer [62], but no data have been reported in mEOC. 

Since mEOC shares several common pathological and molecular features with gastrointestinal (GI) tumors, it has long been hypothesized that standard GI treatments could be more effective for mEOC than the current standard-of-care involving platinum/taxane [11]. mEOC cell lines showed sensitivity to oxaliplatin, etoposide, and 5-fluorouracile (5-FU) as single agents, and the combination oxaliplatin/5-FU appeared to be active. While this translated into improved outcomes in a mouse xenograft model [63], clinical results with these chemotherapy regimens in mEOC are still inconclusive. Indeed, in all the phase I/II cohorts of platinum-refractory EOC patients treated with some of these approaches (e.g., capecitabine, oxaliplatin, FOLFOX, gemcitabine + oxaliplatin), only a very small number of mEOC patients were included, meaning it was difficult to draw clear conclusions. 

Starting from December 2009, the gynecological cancer intergroup (GCIG) made an ambitious but unsuccessful attempt to conduct a specific mEOC trial called GOG241 [64]. The goal of this study was to answer the question as to whether the GI chemotherapy regimen would compare favorably to the standard platinum/taxane doublet approach. This trial also wanted to clarify the efficacy of inhibiting angiogenesis with bevacizumab in mEOC. The aim was to recruit 330 patients with advanced or recurrent mEOC in the first-line setting to be randomized in four arms (83 patients each) to be treated with either carboplatin/paclitaxel ± bevacizumab or oxaliplatin/capecitabine ± bevacizumab. However, only 50 patients had been recruited by early 2013, and the trial was closed. In addition, after a specialist pathological review, some of these patients were actually considered not to have primary mEOC [64]. The partial results from this study were the following: the median progression-free survival (PFS) for the oxaliplatin/capecitabine arms was 10.1 months vs. 15.4 months for the carboplatin/paclitaxel arms (HR 1.08; 95% CI 0.53–2.19; *p* = 0.83). The median PFS in arms with bevacizumab was 17.4 months vs 8.8 months in arms without bevacizumab (HR 0.88; 95% CI 0.43–1.79; *p* = 0.72). Unfortunately, although this latter observation suggests a superior PFS for bevacizumab, the limited number of participants precluded any meaningful conclusions from this trial. 

Thus, even if it is acknowledged that the efficacy of platinum-based therapy is inferior in mEOC as compared to HGSOC, what still need to be defined are the best therapeutic practices due to the lack of adequately powered clinical studies. This means that the chemotherapeutic regimens for mEOC remain controversial, and clinicians have to choose between an inferior standard or a biologically plausible but not yet proven alternative. As recently reported by the American Cancer National Comprehensive Cancer Network (NCCN) guidelines [65] and outlined in [25], mEOC stage I does not need adjuvant treatment as the survival rate is excellent, while stage II–IV mEOC patients should receive adjuvant chemotherapy (3–6 cycles of taxane/carboplatin or 5-fluorouracile/oxaliplatin or capecitabine/oxaliplatin).

### 2.2. Targeted Therapeutic Strategies

In line with the molecular characteristics of mEOC, several targets seem to have potential therapeutic interest for mEOC treatment. The anti-EGFR monoclonal antibody cetuximab exerted anti-proliferative activity only in mEOC cell lines, which did not harbor *KRAS* mutations, and this observation was corroborated in an in vivo murine model, once again mirroring GI malignancies [66]. Clinical trials to support the use of cetuximab in mEOC patients are worth conducting. 

There are very limited clinical data on anti-HER2 therapy in patients with mucinous ovarian tumors. One of these studies investigated HER-2 status in 33 mEOCs and 16 mucinous borderline ovarian tumors. Six of the mEOCs were HER2-positive (18%) and three (19%) of the mucinous borderline ovarian tumors were HER2-positive. Three patients with prospectively determined HER2-positive recurrent mEOC were treated with trastuzumab, and one had a dramatic response to the combination of chemotherapy and trastuzumab [67]. 

Another potential therapeutic opportunity is the targeting of Src kinase. This is a nonreceptor tyrosine kinase which has been implicated in mechanisms of oxaliplatin resistance in colorectal cancer [68]. Encouraging results have been obtained by targeting Src signaling with dasatinib [58] and KX-01, a novel dual Src and anti-tubuline inhibitor in preclinical mEOC models [59]. Considering that the PI3K pathway is reported to be alterated in mEOC, this could be a putative target in mEOC. A dual PI3K and mTOR inhibitor successfully inhibited growth in mEOC cell lines and suppressed tumor growth in in vivo models [69]. Finally, another group recently reported synergistic activity with PI3K/mTOR and MEK inhibition in vivo in mEOC models [70]. Obviously, this preclinical evidence of the potential therapeutic effectiveness of inhibitors of pro-survival pathways in mEOC needs to be investigated further and developed in a clinical setting. 

## 3. Concluding Remarks

mEOC is a subtype of EOC with unique clinical and molecular characteristics. Most patients are diagnosed at an early stage and do not need adjuvant therapy, being cured by surgery alone. However, adjuvant treatment is required for advanced mEOC, though the best therapeutic regimens have not yet been defined. Considering the low incidence of mEOC, the true challenge is the implementation of carefully designed trials that involve, for example, using an adaptive approach to allow for the identification and termination of inactive treatments. Trials will need to be international and funded, involving many cancer centers, and they will have to take into account the unavoidable slow accrual. There is, however, the need to improve mEOC preclinical models, as at the present, very few in vivo preclinical models (i.e., patient-derived xenograft models) and no genetically modified murine models are available [71,72]. An improvement in next-generation sequencing techniques will hopefully lead to a more detailed molecular profiling of mEOC patients to identify possibly pathogenic drivers as targets. High throughput screens of compounds libraries, siRNA libraries, and recently CRISPR/Cas9 libraries could provide a valuable basis for the development of rational, new, therapeutic strategies as single agents and in combinations with compounds targeting those specific pathways found altered in mEOC. 

## Figures and Tables

**Table 1:** Main clinical, molecular, and histopathological features of mEOC and HGSOC.

**Clinical Presentation**			**mEOC**	**HGSOC**
**Diagnosis (% of patients)**	Stage I–II		80%	10%
	Stage III/IV		20%	90%
**OS after platinum-based therapy (No of patients, months)**	Pectasides et al., (2005) [28]	Stage III/IV	47 pts	94 pts
			33.2	38
	Alexandre et al., (2010) [29]	Stage III/IV	54 pts	786 pts
			21.6	47.2
	Bamias et al., (2010) [30]	Stage III/IV	44 pts	367 pts
			14	42
	Karabuk et al., (2013) [31]	Stage III/IV	50 pts	88 pts
			35	94
**Molecular mutations (% of positivity)**			**mEOC**	**HGSOC**
	*P53*		16–52%	99%
	*KRAS*		40–50%	10–22%
	*HER2*		20–30%	none
	*BRAF*		3.5%	none
	*BRCAness phenotype*		none	50%
**Immunohistochemical** **expression markers**			**mEOC**	**HGSOC**
	CK7		+	+
	CK20		+	-
	CEA		+	-
	CA19.9		+	-
	CDX2		+	-
	CA125		-	+
	ER		-/+	+
	DPC4		+	+
	P16		-	-
	WT1		-	+

mEOC: mucinous ovarian cancer; HGSOC: high-grade serous ovarian carcinoma; OS: overall survival; -:negative staining; +: positive staining; -/+; poor staining.
